# Zeaxanthin, a Molecule for Photoprotection in Many Different Environments

**DOI:** 10.3390/molecules25245825

**Published:** 2020-12-10

**Authors:** Barbara Demmig-Adams, Jared J. Stewart, Marina López-Pozo, Stephanie K. Polutchko, William W. Adams

**Affiliations:** Department of Ecology and Evolutionary Biology, University of Colorado, Boulder, CO 80309-0334, USA; jared.stewart@colorado.edu (J.J.S.); marina.lopezpozo@colorado.edu (M.L.-P.); stephanie.polutchko@colorado.edu (S.K.P.); william.adams@colorado.edu (W.W.A.III)

**Keywords:** carotenoid, chlorophyll fluorescence, energy dissipation, lutein, human nutrition, lutein, non-photochemical quenching, photoprotection, violaxanthin, xanthophyll cycle

## Abstract

Conversion of sunlight into photochemistry depends on photoprotective processes that allow safe use of sunlight over a broad range of environmental conditions. This review focuses on the ubiquity of photoprotection associated with a group of interconvertible leaf carotenoids, the xanthophyll cycle. We survey the striking plasticity of this process observed in nature with respect to (1) xanthophyll cycle pool size, (2) degree and speed of interconversion of its components, and (3) flexibility in the association between xanthophyll cycle conversion state and photoprotective dissipation of excess excitation energy. It is concluded that the components of this system can be independently tuned with a high degree of flexibility to produce a fit for different environments with various combinations of light, temperature, and other factors. In addition, the role of genetic variation is apparent from variation in the response of different species growing side-by-side in the same environment. These findings illustrate how field studies can generate insight into the adjustable levers that allow xanthophyll cycle-associated photoprotection to support plant photosynthetic productivity and survival in environments with unique combinations of environmental factors.

## 1. Overview of Sources and Roles of Carotenoids

Carotenoids are responsible for the yellow or orange color of egg yolk [1], corn kernels [2], vegetables and fruits [3,4], and flowers [5,6]. The largest fraction of carotenoids, however, is masked by chlorophyll (see Section 3 and Section 4 below) in photosynthetic organisms. This underlying carotenoid presence in the green foliage of plants is revealed when chlorophyll is degraded and the leaves change color during the autumn in seasonally cold regions [7,8]. Carotenoids support a photosynthetic organisms’ unique task of using sunlight to make food, fuels, and other important molecules under the wide range of different, and continuously changing, light environments encountered in nature. Some of these carotenoids are essential micronutrients for humans. In addition to β-carotene’s role as provitamin A in human vision and immune function, essential roles are emerging for other carotenoids, such as zeaxanthin that is a key factor in vision protection, an anti-inflammatory agent, and also supports brain function and performance of complex mental tasks (for a review, see [9]). Since leaf zeaxanthin level is highly variable and responds strongly to the light environment as well as plant growth rate, it is of interest to define plant growth conditions and species that simultaneously maximize zeaxanthin production and plant productivity. In addition to such environmental manipulation, genetic manipulation of leaf carotenoid level is being explored for the development of more productive and/or resource-efficient crops (see, e.g., [10]). Our review summarizes leaf carotenoid levels’ dynamic adjustment in response to a wide range of natural environments with different combinations of light level, temperature, and other factors. We refer to others for authoritative reviews on carotenoid function in photosynthetic organisms other than plants [11,12,13,14,15].

## 2. Carotenoid Biosynthesis and Biochemical Control of Xanthophyll Cycle Conversions

**Figure 1:**
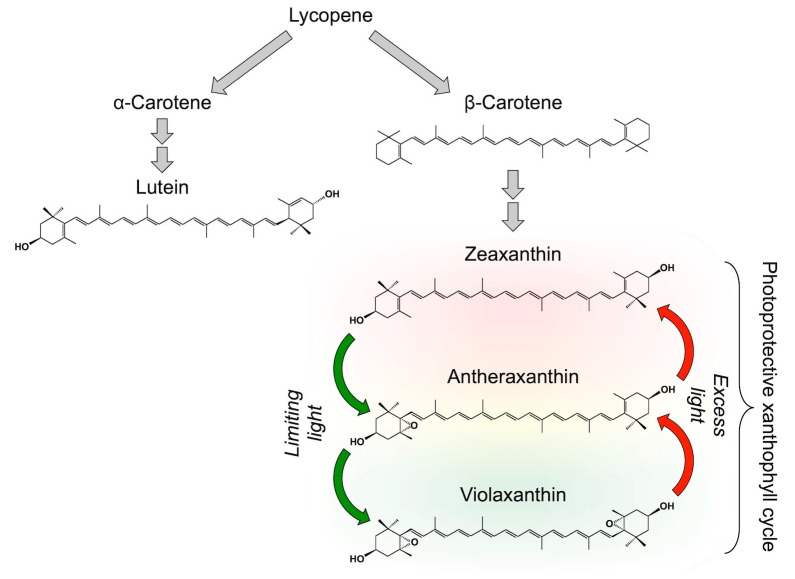
Schematic depiction of the synthesis from lycopene (which does not accumulate in leaves) of foliar carotenoids α-carotene, lutein, β-carotene, and zeaxanthin, as well as interconversions of zeaxanthin, antheraxanthin, and violaxanthin in the xanthophyll cycle.

In leaves, lycopene (a carotene, i.e., oxygen-free carotenoid, that lends the red color to tomatoes) is converted to either α- or β-carotene, each of which is further converted to a corresponding xanthophyll (carotenoid that contains oxygen), specifically lutein and its structural isomer zeaxanthin, respectively (Figure 1). Under low light levels limiting to photosynthesis, zeaxanthin is further converted to violaxanthin (a di-epoxide) via the mono-epoxide intermediate antheraxanthin (Figure 1). The reverse reaction occurs in high light, specifically under light intensities that exceed the level of light that can be consumed by photochemistry. Sapozhnikov et al. [16] were the first to describe a decrease in violaxanthin upon transfer of a leaf from darkness to high light. Harry Yamamoto and colleagues determined that violaxanthin was converted to zeaxanthin [17], and his group and that of Achim Hager [18] identified the enzymes of this cycle and their regulation. Zeaxanthin is converted to violaxanthin by zeaxanthin epoxidase under low-light conditions or in darkness; upon exposure to high light, violaxanthin is de-epoxidized back to zeaxanthin by violaxanthin de-epoxidase. Violaxanthin de-epoxidase activity is triggered when the pH in the internal space of the photosynthetic membranes drops below a certain threshold, which occurs when light absorption exceeds the rate at which ATP, as a key product of the light reactions, can be consumed (see, e.g., [19]). This conversion cycle was initially termed the violaxanthin cycle. Since a special role emerged for zeaxanthin in allowing plants to cope with high light, this cycle is frequently referred to as the xanthophyll cycle, along with other xanthophyll cycles that interconvert xanthophyll epoxides to epoxide-free xanthophylls [17,20,21,22,23].

In his foreword to a review on “In vivo functions of carotenoids in higher plants” [24], Norman Krinsky wrote, “Almost 40 years ago, David Sapozhnikov and his associates from Russia first described the light-induced deepoxidation of the plant carotenoid epoxide, violaxanthin to non-epoxide derivatives, and the reversal of the process in the dark. Since then, many workers have tried to understand the role of the epoxidation/deepoxidation cycle in nature.” When additional layers of tunability began to emerge for this cycle (see Section 6.4 and Section 6.7 below), Harry Yamamato [17] mused, “Why has nature retained this complex, apparently multifunctional system if it is not of some critical advantage?”

## 3. Foliar Carotenoid Levels across Multiple Layers of a Rainforest Canopy

**Figure 2:**
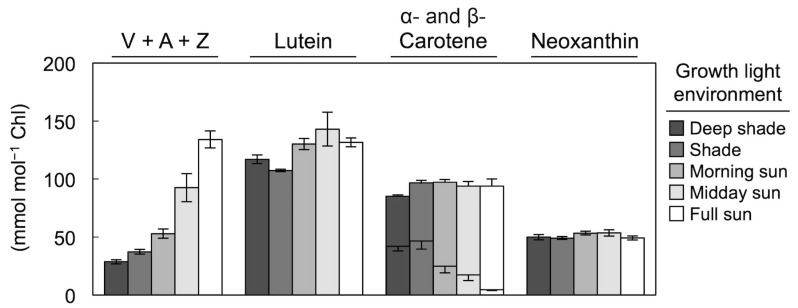
Levels of the primary carotenoids (relative to chlorophyll) present in leaves of 19 species growing in a subtropical rainforest (Dorrigo National Park, New South Wales, Australia) across five light environments from deep shade to full sunlight. Plants growing in Deep shade were never exposed to direct sunlight, those in Shade likely experienced brief periods of high light (sunflecks), those that received Morning or Midday sun were growing where gaps in the canopy permitted direct sunlight to penetrate for longer periods of time, and the most exposed category of plants (Full sun) was at the northern edge of the forest or in the upper canopy where the plants received full sunlight throughout the day. The three carotenoids of the xanthophyll cycle (violaxanthin, antheraxanthin, and zeaxanthin) were pooled together (V + A + Z), as were α-carotene (lower portion of each column) and β-carotene (upper portion of each column). Means ± standard errors are shown. For Deep shade, *n* = leaves from 10 plants from among seven species. For Shade, *n* = leaves from 12 plants from among eight species. For Morning sun, *n* = leaves from seven species. For Midday sun, *n* = leaves from 4 plants from among three species. For Full sun, *n* = leaves from 8 plants from among six species. Data from Logan et al. [25].

Figure 2 shows how the major classes of carotenoids found in leaves respond to a gradient in light intensity across the vertical layers of a subtropical rainforest from deepest shade to full sunlight ([25]; see also [26,27,28,29,30]). The pool of the three interconvertible xanthophylls, violaxanthin, antheraxanthin, and zeaxanthin, increased strongly with increasing light level. Two other xanthophylls, lutein and neoxanthin, did not respond appreciably to the light environment. While the total pool of carotenes also remained similar across this light gradient, there was a pronounced shift in the ratio of α-carotene (prevalent in shade environments) to β-carotene (exhibiting higher levels in full sun).

## 4. Xanthophyll Cycle Conversion State in Response to Growth Light Intensity

**Figure 3:**
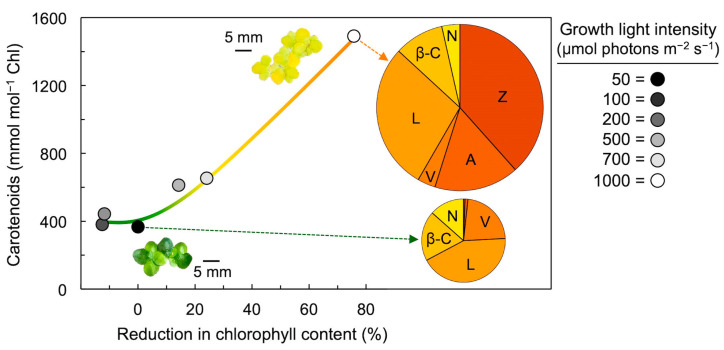
Level of carotenoids (relative to chlorophyll) as a function of reduction in chlorophyll content in fronds of duckweed (*Lemna gibba*) that had developed under continuous light ranging across six growth light intensities from 50 to 1000 µmol photons m^−2^ s^−1^ (i.e., from 4.32 to 86.4 mol photons per square meter per 24-h period). Reduction in chlorophyll content is expressed as percent reduction relative to the chlorophyll level in fronds grown under the lowest light intensity of 50 µmol photons m^−2^ s^−1^. Images of duckweed colonies that developed under 50 and 1000 µmol photons m^−2^ s^−1^ are also shown, as well as pie diagrams depicting the relative levels of each of the six carotenoids present in those fronds (where total pie area represents carotenoid content per chlorophyll). Chl = chlorophyll, V = violaxanthin, A = antheraxanthin, Z = zeaxanthin, L = lutein, β-C = β-carotene, and N = neoxanthin. Data for fronds grown under 100, 200, 500, and 700 µmol photons m^−2^ s^−1^ are derived from Stewart et al. [31]. Data for fronds grown under 50 and 1000 µmol photons m^−2^ s^−1^ are from Stewart et al. [32].

Figure 3 depicts the ratio of carotenoids to chlorophyll in a floating plant with very thin leaves—and, consequently, very little internal self-shading—when grown experimentally under continuous light of intensities ranging from low to very high, with a maximal total daily photon flux slightly higher than that on the brightest longest day on Earth [31,32]. As chlorophyll levels declined with increasing growth light, carotenoids continued to accumulate, leading to an exponential increase in the levels of carotenoids relative to chlorophyll. In addition, the composition of the carotenoid pool is shown for the lowest (green plants) and the highest (yellow plants) growth light intensities, with the size of the pie representing the relative total carotenoid to chlorophyll level. While the entire xanthophyll cycle pool was present as violaxanthin under 50 µmol photons m^−2^ s^−1^, the majority of this pool was represented by zeaxanthin under very high light, with the majority of the remainder present as antheraxanthin.

## 5. Leaves Track Changes in Light Level over the Course of the Day with Adjustments in Xanthophyll Cycle Conversion State

**Figure 4:**
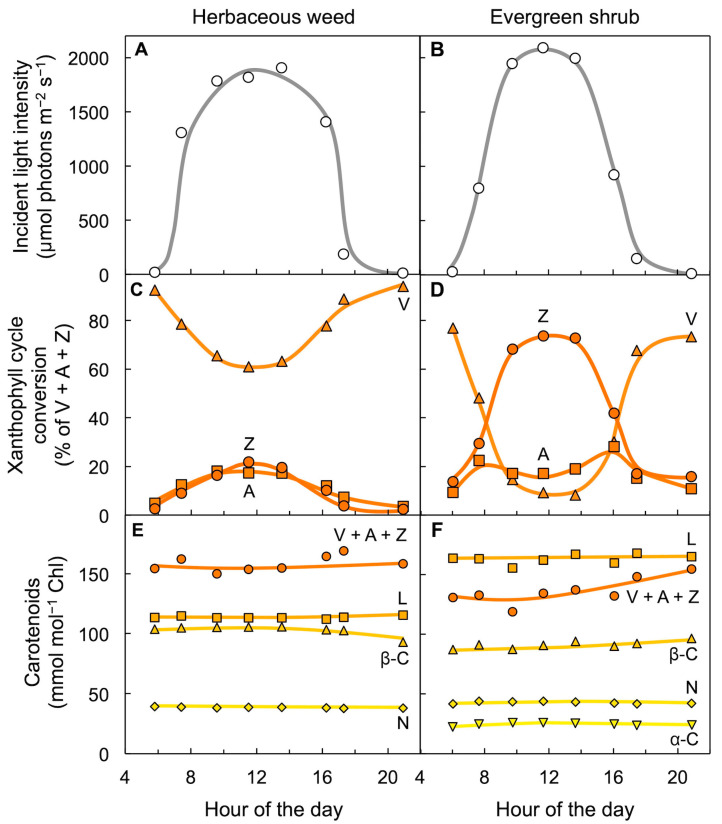
Diurnal characterization of foliar carotenoid content for leaves of an herbaceous, biennial weed (*Malva neglecta*) that tracks the sun and a woody, evergreen shrub (*Euonymus kiautschovicus*) on a sunny day (15 September 1990). (**A**,**B**) Direct sunlight incident on the upper leaf surface, (**C**,**D**) conversion state of the xanthophyll cycle with each component (V = violaxanthin, Z = zeaxanthin, and A = antheraxanthin) expressed as percent of total xanthophyll cycle pool (V + A + Z), and (**E**,**F**) levels of α-carotene (α-C), neoxanthin (N), β-carotene (β-C), lutein (L), and total xanthophyll cycle pool (V + A + Z) relative to chlorophyll (Chl). Data are from Adams and Demmig-Adams [33].

Figure 4 shows foliar carotenoid levels in two plant species growing side-by-side in full sunlight, one a fast-growing weed, the other a slow-growing evergreen [33]. Fast-growing species have higher photosynthetic capacities than slow-growing species [33,34,35], which also corresponds to higher maximal rates of photosynthetic electron transport in fast-growing compared to slow-growing species [36]. Sun-exposed leaves of both species formed considerable amounts of zeaxanthin every day as the light intensity incident on leaves rose from morning to midday, and then reconverted zeaxanthin to violaxanthin in the afternoon as incident light levels declined again (Figure 4A–D). However, the evergreen with its lower rate of light utilization in photosynthetic electron transport converted a much greater percentage of the total xanthophyll cycle pool to zeaxanthin than the herbaceous weed (Figure 4C,D; for a review, see [37]; for a comparison of evergreen versus drought-deciduous oaks, see [38]). These field observations clearly indicate that xanthophyll cycle conversions follow light level on a daily basis in high-light environments—even in fast-growing plants that utilize much of the harvested light for photosynthesis and growth. The greater extent of zeaxanthin formation in the slow-growing evergreen compared to the fast-growing weed in the same light environment is consistent with a role of zeaxanthin in plant response to the unused portion of light—the fraction not consumed in photochemistry. In contrast to the three xanthophylls of the xanthophyll cycle, all other carotenoids (ubiquitously present lutein, neoxanthin, and β-carotene as well as carotenoids not present in all systems, such as α-carotene (Figure 4F) and lactucaxanthin (not shown) in *E. kiautschovicus*) remained unchanged over the course of the day despite the pronounced changes in light level striking the leaf (Figure 4E,F). One may ask whether evergreens grow more slowly because they are less efficient at using absorbed light or whether their slower growth rate limits how much light they can utilize at peak irradiance. The fact that the maximal photosynthesis rate is subject to control by demand from the rest of the plant for photosynthates, and especially the rate of sugar export from leaves [39,40], suggests that a lower demand for the products of photosynthesis in the slow-growing evergreen decreases the fraction of light absorbed at peak irradiance that is available for utilization in photosynthesis.

**Figure 5:**
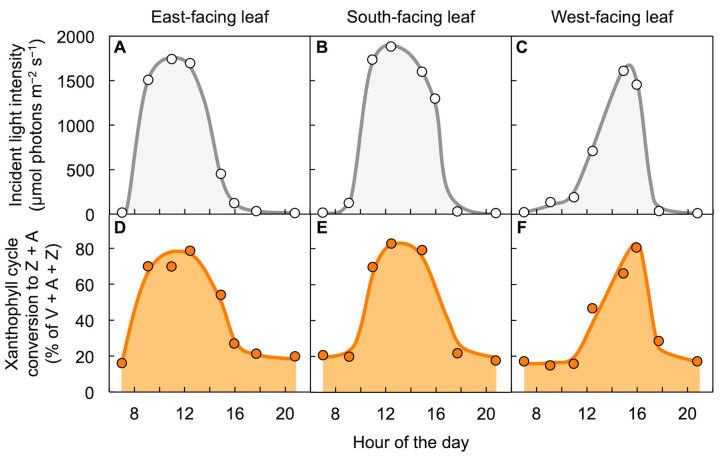
Diurnal characterization of the conversion state of the xanthophyll cycle in east-, south-, and west-facing leaves of *Euonymus kiautschovicus* on a sunny day (25 October 1990). (**A**–**C**) Direct sunlight incident on the upper leaf surfaces and (**D**–**F**) percent of the xanthophyll cycle converted to zeaxanthin and antheraxanthin (Z + A). V = violaxanthin. Data are from Adams et al. [41].

Field characterization of the evergreen with different leaf orientations confirmed, and expounded on, these findings [41]. East-facing leaves that received peak levels of sunlight in the morning reached their maximal zeaxanthin levels earlier in the morning (Figure 5A,D); south-facing leaves receiving peak sunlight closer to midday exhibited peak zeaxanthin leaves at midday (Figure 5B,E); and west-facing leaves experiencing peak light level in the afternoon reached peak zeaxanthin levels later in the day (Figure 5C,F). Each leaf thus formed the most zeaxanthin when experiencing the greatest levels of unutilized light and re-converted zeaxanthin to violaxanthin as light levels declined again over the course of a day. These findings illustrate the importance of paying close attention to orientation/exposure and incident light level in studies of xanthophyll cycle operation (see also [42,43,44,45]).

## 6. Xanthophyll Cycle Conversion State and Dissipation of Unused Light as Thermal Energy

### 6.1. Harmless Dissipation of Unused Light: The Non-Photochemical Route to Thermal Energy

**Figure 6:**
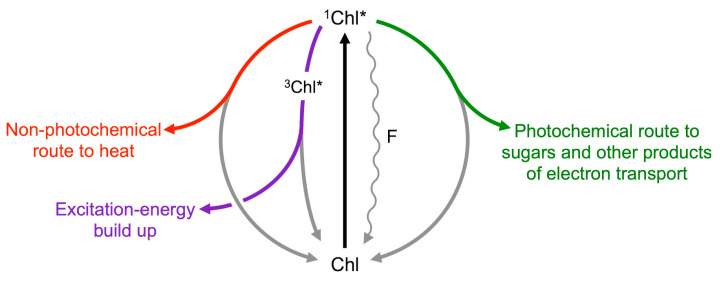
Possible fates of excitation energy once a photon of light is absorbed by a molecule of chlorophyll resulting in conversion from the ground state (Chl) to the singlet excited state (^1^Chl*). When light is not saturating for photosynthesis, the majority of chlorophyll molecules pass their excitation energy to neighboring chlorophyll molecules via resonance transfer (resulting in return of the first chlorophyll molecule to its ground state). This process can be repeated until the energy reaches a reaction center chlorophyll molecule that undergoes charge separation, resulting in an electron entering the photosynthetic electron transport chain (photochemical route denoted in green, returning the reaction center chlorophyll molecule to its ground state). As light levels become increasingly excessive, excess excitation energy is increasingly dissipated preemptively as thermal energy via a regulated pathway (non-photochemical route denoted in red that also returns the excited chlorophyll molecule to its ground state; see text for regulation of the xanthophyll cycle and engagement of zeaxanthin in energy dissipation). When the combined levels of photochemical and regulated non-photochemical routes are insufficient to swiftly de-excite all excited singlet chlorophyll, conversion to triplet excited chlorophyll (^3^Chl*) takes place, which represents a temporary build-up of excess excitation energy (purple route). In the presence of molecular oxygen, triplet excited chlorophyll returns to its ground state by transfer of excitation energy to oxygen, resulting in the formation of singlet excited oxygen (not shown). A small percentage of the chlorophyll molecules return to ground state directly from the singlet excited state (wavy line), emitting fluorescence (F). Although only representing a very small fraction of the excitation energy, and thus not a major route for deexcitation, fluorescence emission provides valuable quantitative information about the other fates (particularly the regulated non-photochemical and photochemical routes).

The foreword from Norm Krinsky [24] referenced above continues, “Only in the last few years, however, has evidence accumulated that the deepoxidation products, such as zeaxanthin, play a significant role in dissipating light energy that is not used directly for photosynthesis.” Figure 6 illustrates how chlorophyll fluorescence can be used to track the fate of absorbed light as excitation energy that is available for utilization through a photochemical route for photosynthetic electron transport or, alternatively, dissipated harmlessly and preemptively as thermal energy through a highly regulated non-photochemical route. Photochemical routes for excitation energy include not only carbon fixation for sugar production but also photorespiration, reduction of nitrite and other nutrients, and the water-to-water cycle of the Mehler reaction [46].

Transient build-up of any excitation energy not promptly removed via either photochemical or regulated non-photochemical routes results in formation of singlet oxygen, which can trigger cascades ranging from (i) production of signals that upregulate evasive responses to (ii) inactivation of the photochemical reactions and, eventually, (iii) programmed cell death (for a review, see [47]). Singlet oxygen formation technically also dissipates energy non-photochemically but is not a regulated pathway. Similarly, chlorophyll fluorescence is technically a de-excitation pathway, but of negligible capacity, and provides information on the respective volumes of excitation energy flow through the other pathways (Figure 6; [31,46]). As shown in Section 6.7 below, the extent of regulated non-photochemical dissipation of excitation as thermal energy can be assessed from non-photochemical fluorescence quenching or, under many conditions, from the decrease in the fraction of absorbed light that is available for utilization in photochemistry.

### 6.2. Dissipation of Unused Light as Thermal Energy Tracks Xanthophyll Cycle Conversions in Sun-Exposed Plants under Otherwise Favorable Condition

**Figure 7:**
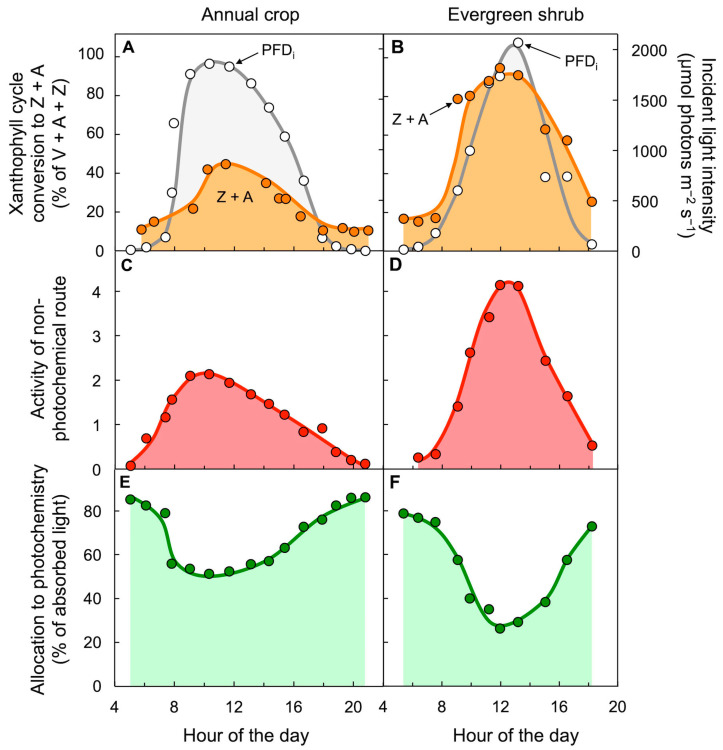
Diurnal characterization of (**A**,**B**) light incident on the upper leaf surface (PFD_i_) and percent of the xanthophyll cycle present as zeaxanthin and antheraxanthin (Z + A), (**C**,**D**) dissipation of excitation energy as thermal energy via a regulated non-photochemical route (quantified from chlorophyll fluorescence as Stern–Volmer quenching), and (**E**,**F**) percentage of absorbed photons allocated to photochemistry (quantified from chlorophyll fluorescence as photosystem II efficiency) in leaves of the herbaceous annual sunflower (**A**,**C**,**E**) and the woody evergreen *Euonymus kiautschovicus* (**B**,**D**,**F**) on summer days. V = violaxanthin. Data for sunflower are from Demmig-Adams and Adams [48] and Demmig-Adams et al. [49]. Data for *E. kiautschovicus* are from Verhoeven et al. [50].

**Figure 8:**
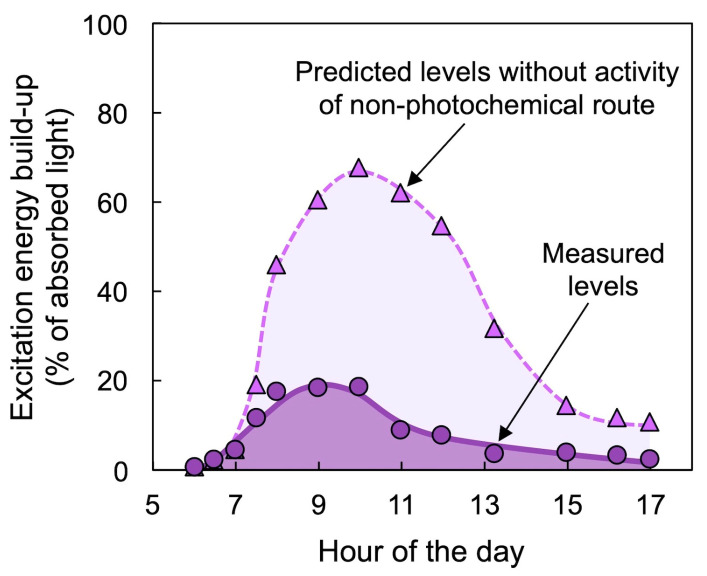
Levels of measured (quantified from chlorophyll fluorescence as photosystem II reduction state; purple circles) and predicted (calculated for the absence of non-photochemical energy dissipation; purple triangles) build-up of excess excitation energy over the course of a day in the east-facing surface of a cactus (*Nopalea cochenillifera*) cladode. Data are from Demmig-Adams and Adams [19].

Figure 7 shows that conversion of the xanthophyll cycle pool to the de-epoxidized forms Z + A (Figure 7A,B) was closely correlated with dissipation of unused light as thermal energy, as assessed from either non-photochemical fluorescence quenching (a measure of the extent of excitation energy dissipated preemptively through the regulated non-photochemical route; Figure 7C,D) or from the decreased percentage of excitation energy that is available for utilization in photochemistry (Figure 7E,F) [48,49,50]. These latter two measures are mirror images of each other (Figure 7C–F) when thermal energy dissipation is a significant contributor to the decreased ability to use excitation energy for photochemistry. Figure 8 shows the hypothetical much greater transient increase in excitation energy build-up (increased reduction state of photosystem II centers) in the calculated absence of thermal energy dissipation, as obtained from chlorophyll fluorescence parameters. It should be noted that the toggle switch of the photosynthetic apparatus to a state of strong preemptive thermal energy dissipation represents efficient competition of the regulated non-photochemical route with the photochemical route, which effectively prevents excitation energy build-up but also requires careful control of this switch to ensure the return to efficient light utilization in photosynthesis whenever possible.

As an alternative to thermally dissipating large amounts of unused absorbed light energy at peak irradiance, a leaf could produce a lot less chlorophyll. This would, however, diminish the leaf’s ability to collect light efficiently during long low-light periods of the day in the morning and afternoon. Preemptive removal of unusable light at midday with the help of zeaxanthin offers the advantage of allowing the leaf to have enough chlorophyll for harvesting light efficiently during periods when light is sub-saturating for photosynthesis, while protecting this chlorophyll effectively during peak irradiance via facile biochemical xanthophyll conversions rather than deconstruction and reconstruction of light-harvesting complexes. Likewise, removal of the dissipater zeaxanthin in the morning and afternoon presumably helps to safeguard against undesirable removal of excitation energy that is usable in photosynthesis under limiting light levels. Only the xanthophyll cycle carotenoids follow the light level, while all other carotenoids remain at similar levels all day. As further addressed in Section 6.4 and Section 6.7 below, there are additional mechanisms at play that engage and disengage zeaxanthin in the act of thermal energy dissipation as an additional layer of control over the critical fate of the excitation energy (see, e.g., [14,51]). These mechanisms of engagement and disengagement offer additional flexibility in plant response to multiple different combinations of environmental challenges.

The photophysical mechanism of how excitation energy is removed from excited chlorophyll was under debate for decades (see edited volume by Demmig-Adams et al. [52]) after the initial proposal of a link between zeaxanthin and the dissipation of excitation energy as heat [53,54,55,56,57,58,59]. Polivka and Frank [60] elaborated on the difficulties of studying these mechanisms due to the impact of the microenvironment on the photophysical properties of photosynthetic pigments. Since then, Graham Fleming’s group conducted fluorescence lifetime studies in vivo in an intact photosynthetic organism and concluded that zeaxanthin was involved in both of the two different photophysical mechanisms (energy transfer and charge transfer) capable of de-exciting chlorophyll thermally [61,62]. The last portion of the foreword from Krinsky (in [24]) addresses parallels between a leaf and a human eye that accumulates dietary zeaxanthin: “whether a similar process occurs in the macula area of the primate retina, where zeaxanthin is concentrated, is not known.”

Much attention has since been given to the functions of both zeaxanthin and lutein in (i) protecting the human retina from damage by high light (for a review, see [63]) as well as (ii) serving as anti-inflammatories opposing systemic chronic inflammation and (iii) also enhancing visual and mental acuity [9]. These roles in humans and other animals may be catalyzed mainly through the action of zeaxanthin and lutein as antioxidants that counteract lipid peroxidation and as membrane stabilizers that enhance membrane function [9]. In addition to its role in dissipating excess excitation as thermal energy in photosynthesis, zeaxanthin serves as an antioxidant in photosynthetic organisms, counteracting lipid peroxidation, and stabilizes membranes [64,65]. These roles outside of light-collecting protein complexes appear to be carried out predominantly by zeaxanthin in plants, whereas both zeaxanthin and lutein appear to be involved in animals, albeit with a more potent role of zeaxanthin [9]. Future research is needed to examine whether this difference is related to any unique functions of lutein in animal membranes [66] or simply to the scarcity of zeaxanthin compared to lutein in the human diet, which is related to the removal of zeaxanthin in low light as opposed to the constant high lutein levels in the leaves of plants [9].

### 6.3. Light Gradients from Shade to Sun Determine not only Xanthophyll Cycle Dynamics but also Thermal Energy Dissipation Capacity

**Figure 9:**
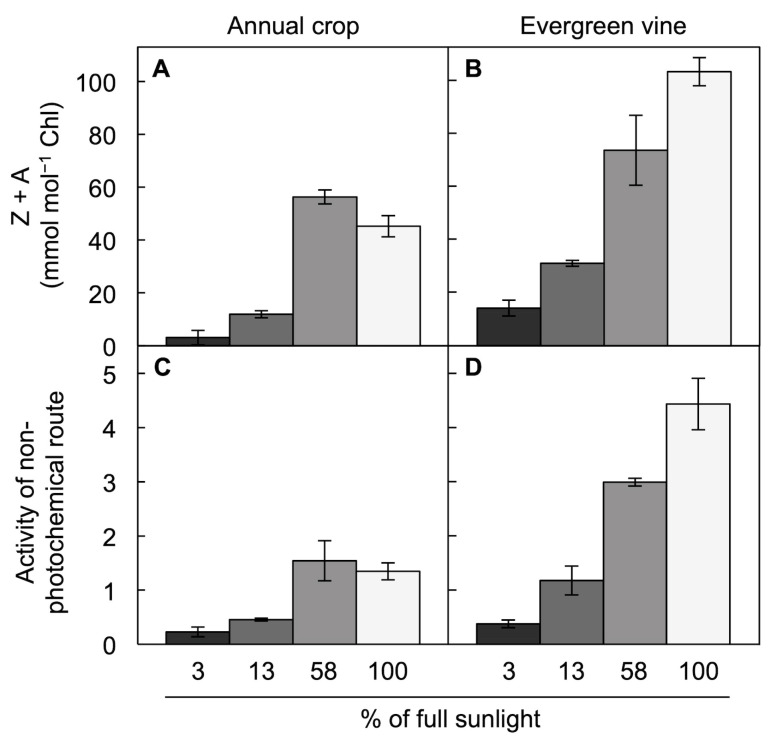
Foliar levels of (**A**,**B**) zeaxanthin + antheraxanthin (Z + A) per chlorophyll (Chl) and (**C**,**D**) energy dissipation via the non-photochemical route (quantified from chlorophyll fluorescence as Stern–Volmer quenching) at midday during the summer in the herbaceous annual pumpkin (**A**,**C**) and the evergreen groundcover *Vinca major* (**B**,**D**). Plants were grown in the field under a shade cloth, resulting in penetration of 3% of full sunlight (black columns), 13% of full sunlight (dark gray columns), or 58% of full sunlight (medium gray columns), or with no shade cloth (100% or full sunlight; light gray columns). Mean values ± standard deviations, *n* = three leaves. Data are from Logan et al. [36].

In addition to the size of the xanthophyll cycle pool ([25,26,27,28]; Figure 2), the extent of conversion of this pool to zeaxanthin and antheraxanthin and the fraction of absorbed light dissipated via the regulated non-photochemical route are strongly affected by the light environment and also differ among species (Figure 9; [36]). Leaves growing in the shade (e.g., 60 µmol photons m^−2^ s^−1^ at 3% of full sun in Figure 9) form little zeaxanthin and antheraxanthin at midday, whereas higher levels are formed in leaves that receive more light (Figure 9A,B). This effect of the light environment is even more notable in a slow-growing evergreen (Figure 9B) compared to a fast-growing annual (Figure 9A). The levels of energy dissipation via the regulated, preemptive non-photochemical route (Figure 9C,D) largely parallel zeaxanthin + antheraxanthin levels (Figure 9A,B), from little in low light to moderate levels in the annual (Figure 9A,C) or to high levels in the evergreen (Figure 9B,D) in full sun (see also [67]).

### 6.4. Thermal Energy Dissipation Activity Changes More Rapidly than Xanthophyll Cycle Conversions in Fluctuating Light Environments

**Figure 10:**
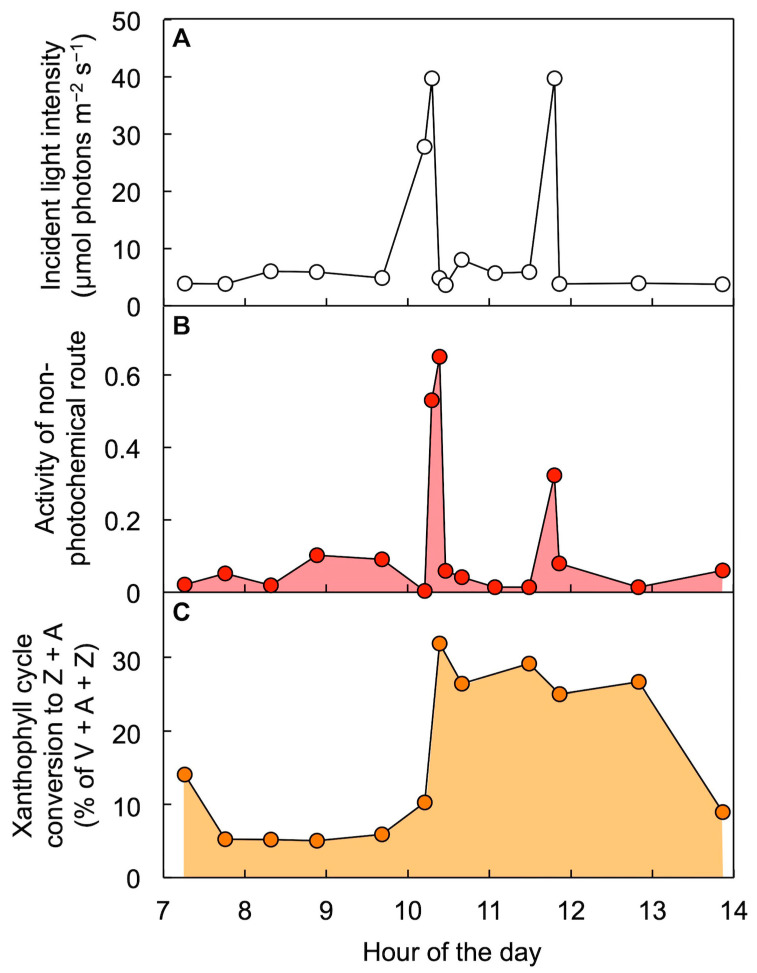
Diurnal characterization of (**A**) light incident on the upper leaf surface, (**B**) level of energy dissipation via a regulated non-photochemical route (quantified from chlorophyll fluorescence as Stern–Volmer quenching), and (**C**) percent of the xanthophyll cycle pool present as zeaxanthin + antheraxanthin (Z + A) in leaves of *Alocasia brisbanensis* in the understory of a subtropical rainforest (Dorrigo National Park, New South Wales, Australia). V = violaxanthin. Data are from Logan et al. [68].

**Figure 11:**
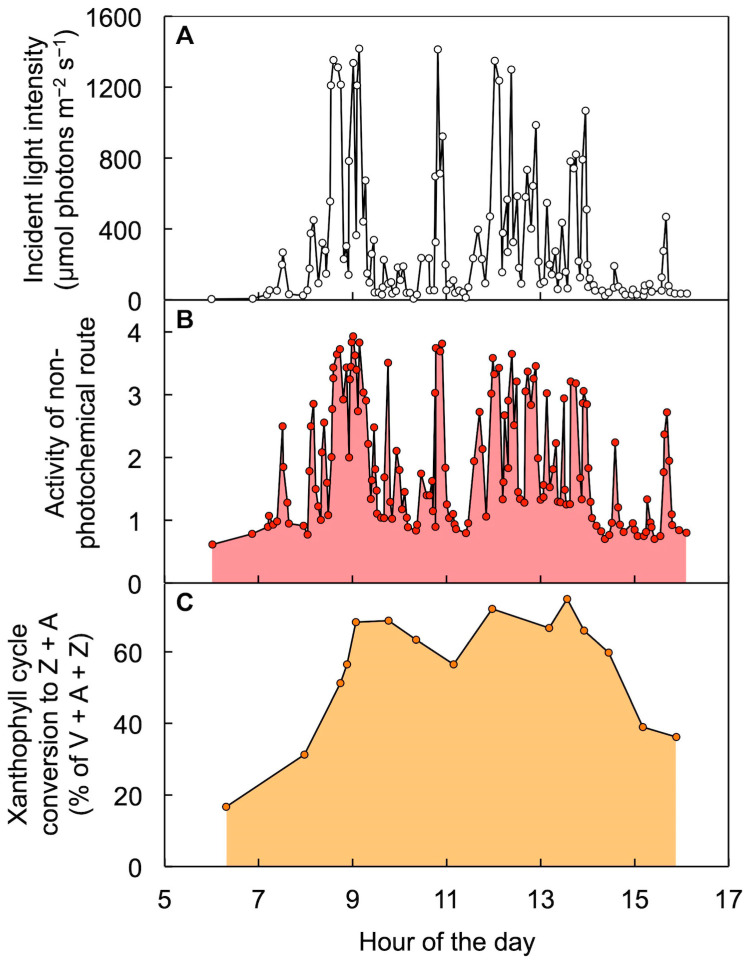
Diurnal characterization of (**A**) light incident on the upper leaf surface, (**B**) level of energy dissipation via a regulated non-photochemical route (quantified from chlorophyll fluorescence as Stern–Volmer quenching), and (**C**) percent of the xanthophyll cycle pool present as zeaxanthin + antheraxanthin (Z + A) in leaves of *Stephania japonica* in the understory of an open Eucalyptus forest (Middle Head, New South Wales, Australia). V = violaxanthin. Data are from Adams et al. [69].

**Figure 12:**
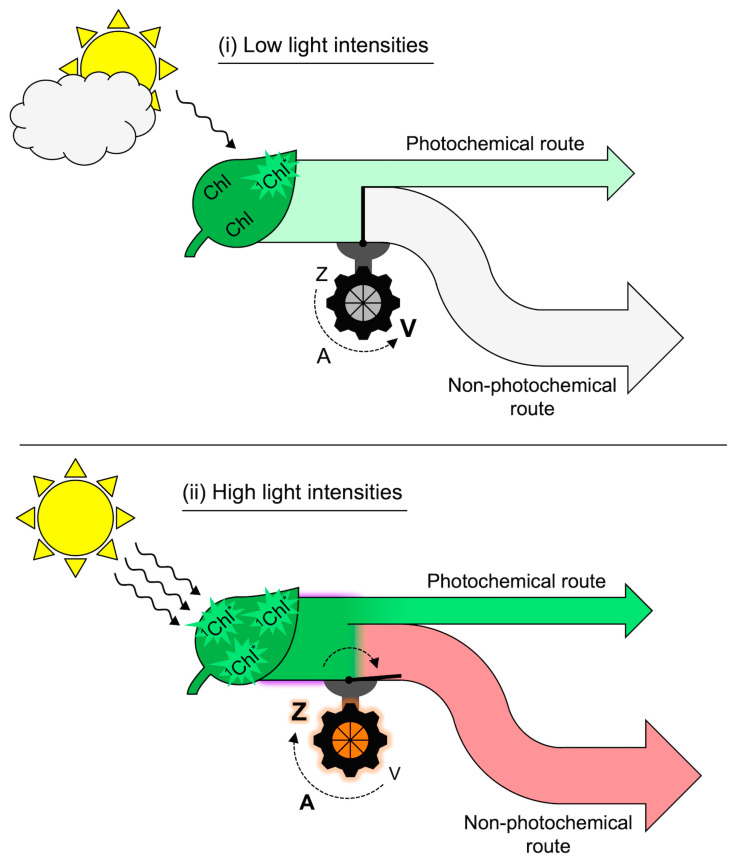
Schematic depiction of (**i**) absorption of low levels of light by chlorophyll (Chl) and allocation of that excitation energy to photochemistry (light green arrow) coupled with conversion of zeaxanthin (Z) to violaxanthin (V) through the intermediate antheraxanthin (A) and closure of the route to regulated non-photochemical energy dissipation versus (**ii**) absorption of high levels of light by chlorophyll and allocation of more excitation energy to photochemistry (dark green flux) coupled with conversion of violaxanthin (V) to zeaxanthin (Z) through the intermediate antheraxanthin (A) and opening of the path to regulated non-photochemical energy dissipation, resulting in a large fraction of excitation energy dissipated as thermal energy (light red arrow). ^1^Chl* = singlet excited-state chlorophyll.

Figure 10 and Figure 11 illustrate responses of leaves to sunflecks, shafts of sunlight penetrating through forest canopies that produce rapid changes in incident light intensity striking leaves in the forest understory [68,69]. In the case of *Alocasia brisbanensis*, growing in the deep shade of a multi-layer rainforest canopy, only two low-intensity sunflecks hit the leaves on this particular winter day, producing rapid increases in the extent of excitation energy dissipated through the regulated non-photochemical route (Figure 10). The xanthophyll cycle became converted to Z + A during the first sunfleck and this elevated Z + A level was maintained up to, and beyond, the second sunfleck of the day. It was as if the leaves anticipated that another sunfleck was coming, which is not far-fetched since the occurrence of sunflecks in the understory of a forest is a highly regular event from day to day. This is because the canopy structure changes very slowly and the position of the sun in the sky shifts minimally from day to day as Earth progresses in its annual orbit. A similar pattern was seen in the understory of an open eucalypt forest with vertically suspended leaves and thus more frequent, high-intensity sunflecks. The vine *Stephania japonica* growing on the forest floor underwent a series of many rapid, pronounced increases and decreases in the extent of regulated non-photochemical energy dissipation over the course of the day, while Z + A level increased with the first sunflecks and remained elevated over the course of the day (Figure 11). This finding clearly demonstrates that the mere presence of zeaxanthin is not sufficient to prompt thermal dissipation of excitation energy and that a second condition is required. Figure 12 illustrates this dual control consisting of (i) zeaxanthin formation in the xanthophyll cycle and (ii) engagement of zeaxanthin in the thermal dissipation of excess excitation energy. Figure 12 also acknowledges that fluctuating light environments are not restricted to the understory of a forest but occur whenever clouds cause the light level incident on leaves to vary dramatically (see [70]).

**Figure 13:**
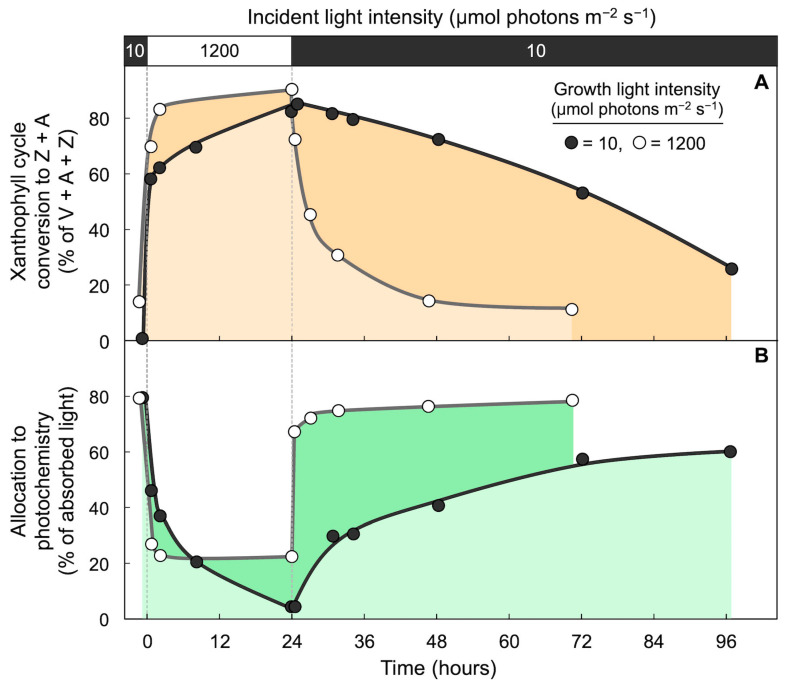
Impact of sudden transfer from low light (10 µmol photons m^−2^ s^−1^) to high light (1200 µmol photons m^−2^ s^−1^; open bar across top) for 24 h followed by return to low light (black bar across top) on leaves of *Schefflera arboricola* that developed in either low light (dark circles) or in high light in a greenhouse where leaves received a peak photon flux density of 1200 µmol photons m^−2^ s^−1^ (open circles). (**A**) Percent of xanthophyll cycle converted to zeaxanthin + antheraxanthin and (**B**) percentage of absorbed photons allocated to photochemistry (quantified from chlorophyll fluorescence as photosystem II efficiency). V = violaxanthin. Data are from Demmig-Adams et al. [73].

A key mechanism that catalyzes rapid engagement and disengagement of thermal energy dissipation involves specialized members of the family of light-harvesting proteins, such as the PsbS protein in plants (for a review, see [51]). A PsbS-deficient *Arabidopsis thaliana* mutant that lacks rapid changes in thermal energy dissipation did not exhibit any appreciable effect on plant productivity in constant high light under controlled conditions, where *A. thaliana* used a large fraction of excitation energy via the photochemical route [71]. In contrast, this PsbS-deficient mutant exhibited impaired function, including reduced seed production, when grown in an outdoors environment with highly fluctuating light [72]. These findings indicate that rapid engagement and disengagement of zeaxanthin in thermal energy dissipation is of particular importance in specific environments, such as rapidly fluctuating light levels. It should be noted that the ability of plants to maintain rapidly reversible control of thermal energy dissipation in high-light environments requires plant acclimation to excess-light conditions, i.e., that leaves must have developed under these excess-light conditions. Plants grown in constant low light, on the other hand, can be considered excess light-naïve. Excess light-naïve evergreens were slow in forming zeaxanthin upon a first exposure to high light [58]. When subjected to prolonged exposure to high light, such plants slowly assumed and then continuously maintained high levels of both thermal energy dissipation and Z + A for extended time periods after the return to low light, which strongly reduced the percentage of absorbed light that can be used for photochemistry under these light-limiting conditions (Figure 13; [73]). In other words, sudden high-light exposure drove low light-grown *Schefflera arboricola* into a state with a high Z + A level and a low percentage of absorbed light available for utilization in photochemistry as is also seen under high-light exposure of sun-grown leaves—except that only the low light-grown leaves were unable to exit this state for days upon the return to low light, while sun-grown leaves of the same species rapidly exited this state upon the return to low light [73].

### 6.5. Environmental Factors other than Light Can Dominate the Response: Mineral Nutrients

**Figure 14:**
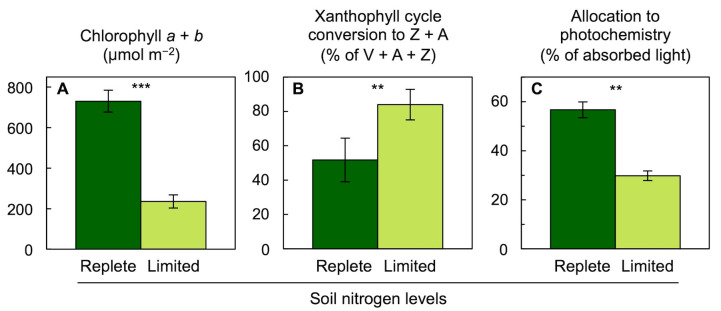
Impact of growth with replete (dark green columns) or limiting (light green columns) soil nitrogen levels on (**A**) chlorophyll content, (**B**) percent of the xanthophyll cycle converted to zeaxanthin + antheraxanthin (Z + A), and (**C**) percentage of absorbed photons allocated to photochemistry (quantified from chlorophyll fluorescence as photosystem II efficiency) in leaves of spinach grown in a naturally lit greenhouse. V = violaxanthin. Means ± standard deviations. Asterisks denote statistically significant differences between soil nitrogen levels (Student’s *t*-test; ** = *p* < 0.01, *** = *p* < 0.001). Data are from Logan et al. [74].

As stated above, plants growing in sunny environments under otherwise favorable conditions maintained high chlorophyll levels and disposed of excess excitation energy at peak irradiation. An annual plant such as spinach grown under nitrogen-deficient conditions that strongly depressed plant growth exhibited pronounced decreases in foliar chlorophyll content (Figure 14A; [74]), presumably as a consequence of downregulation of the synthesis of light-harvesting complexes [75]. At the same time, the conversion state of the xanthophyll cycle to Z + A was increased (Figure 14B) and the percent of absorbed light available for utilization in photochemistry at peak irradiance was decreased (Figure 14C). This decrease in the percentage of absorbed light that can be converted to photochemistry was fully reversed upon darkening of the leaves, suggesting that chlorophyll content was lowered sufficiently to avoid any lasting effects [76], such as an inability to exit the state of low utilization efficiency of excitation energy in photochemistry seen in excess light-naïve *S. arboricola* leaves in the previous section. For the effect of deficiencies in other essential nutrients, see [77].

### 6.6. Environmental Factors other than Light Can Dominate the Response: Seasonal Cold

**Figure 15:**
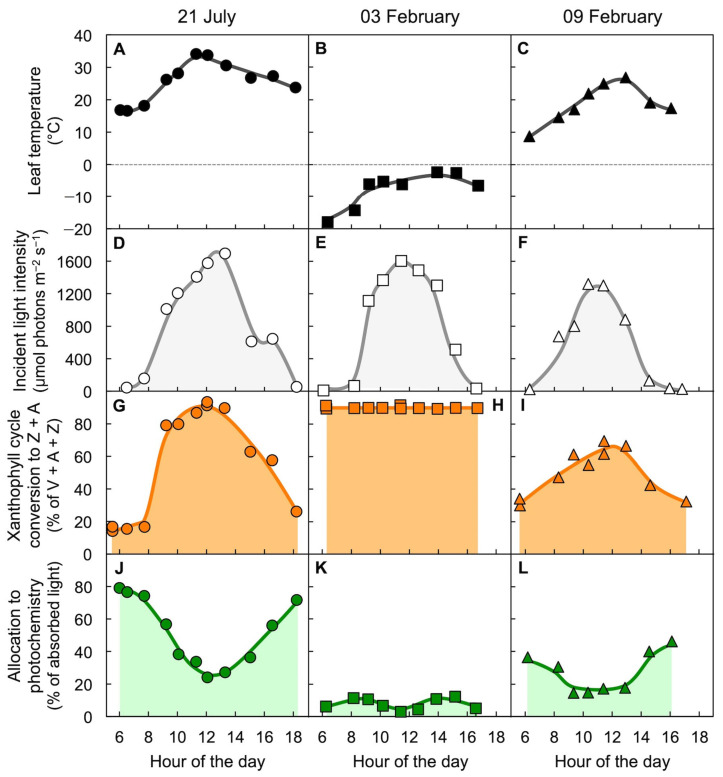
Diurnal characterization of (**A**,**B**,**C**) leaf temperature, (**D**,**E**,**F**) light incident on the upper leaf surface, (**G**,**H**,**I**) percent of the xanthophyll cycle present as zeaxanthin and antheraxanthin (Z + A), and (**J**,**K**,**L**) percentage of absorbed photons allocated to photochemistry (quantified from chlorophyll fluorescence as photosystem II efficiency) in leaves of the woody evergreen *Euonymus kiautschovicus* on a warm day during summer (**A**,**D**,**G**,**H**; circles), on a cold day in winter (**B**,**E**,**H**,**I**; squares), and a subsequent warmer winter day (**C**,**F**,**I**,**L**; triangles). V = violaxanthin. Data are from Verhoeven et al. [50].

Figure 15 shows the air temperature, level of incident light, xanthophyll cycle conversion state, and the percent of absorbed light available for utilization in photochemistry in an overwintering evergreen shrub [50]. Three sample days are shown, a warm summer day in July (Figure 15A), a very cold winter day in February (Figure 15B), and a winter day six days later with mild temperatures (Figure 15C) during a transiently warmer period. On all three days, incident light patterns and maximal light levels were similar (Figure 15D–F). On the summer day, xanthophyll cycle conversion state increased and decreased (Figure 15G), paralleled by a decrease and increase in the percentage of excitation energy available for utilization in photochemistry (Figure 15J) over the course of the day, as shown earlier (Figure 4, Figure 5 and Figure 7). In contrast, on the cold winter day with subfreezing temperatures, maximal conversion of the xanthophyll cycle to Z + A was maintained throughout the day and night (Figure 15H), i.e., on the subfreezing day leaves remained locked in the state they only briefly assumed at peak irradiance on the summer day. This continuously maintained high xanthophyll cycle conversion state was associated with a continuously minimal percentage of absorbed light available for utilization in photochemistry (Figure 15K). Under these conditions, chlorophyll fluorescence remained highly quenched, which does not allow quantification of the activity of the regulated non-photochemical route since that would require a control level of chlorophyll fluorescence in the fully unquenched state [78,79]. Just six days later on a transiently warm winter day, xanthophyll cycle conversion state (Figure 15I) and the percentage of light available for utilization in photochemistry (Figure 15L) were intermediate early in the morning. This result indicates partial, albeit not complete, relaxation of the locked-in high-light state of the xanthophyll cycle and low photochemical energy utilization on the mild winter day. Such dynamic patterns are typically observed in locations where soils do not freeze for prolonged periods in the winter. In contrast, in a high-altitude, subalpine environment where soil water freezes for the duration of the winter, overwintering conifers and other evergreens maintained a state with continuously maximal xanthophyll cycle conversion to Z + A and negligible availability of absorbed light for utilization in photochemistry throughout the entire winter season [37,79,80,81,82,83,84]. In the spring, these evergreens reverted to low predawn xanthophyll cycle conversion and high utilization of absorbed light in the morning and afternoon within a few days as the soil water thawed [85].

This trend to remain in a state of high Z + A and low utilization efficiency of absorbed light for photochemistry for the duration of an entire season, i.e., the ability to lock in a state of extreme photoprotection, is thought to be a prerequisite for maintaining evergreen status (with green leaves) throughout a whole season with extreme conditions [35,37,86]. This scenario in overwintering evergreens—long-term maintenance of high Z + A and low utilization of absorbed light—is reminiscent of the response of low light-grown *S. arboricola* suddenly exposed to high light (see above). Unlike high light-grown plants of the same species (and unlike understory plants growing in rapidly fluctuating light environments), low light-grown *S. arboricola* plants abruptly exposed to high light did not quickly resume efficient photochemical utilization of absorbed light for photosynthesis upon the return to low light. It should be noted that low light-grown evergreens subjected to sudden transfer to high light exhibited pronounced foliar starch build-up rather than carbohydrate-depleted leaves [39,87,88], which suggests that plant growth rate and carbohydrate consumption by the plant’s sinks takes time to increase upon sudden transfer to high light. In overwintering subalpine species, sustained lowering of the fraction of absorbed light available for utilization in photochemistry is of no cost to growth as long as growth is actively suspended in the winter. Rather, the ability to lock in this highly protected state presumably allows subalpine coniferous forests to maintain green leaves that quickly resume activity and perform 90% of their annual carbon gain in the month after soil water thawing and snow melt, when water availability for growth is maximal [89]. This is another example for the apparent adaptive value of the biochemical photoprotection offered by the xanthophyll cycle (see also [38,90,91,92])—as opposed to deconstruction and reconstruction of light-harvesting complexes, or rather, deconstruction and rebuilding of whole leaves in winter-deciduous compared to evergreen species.

**Figure 16:**
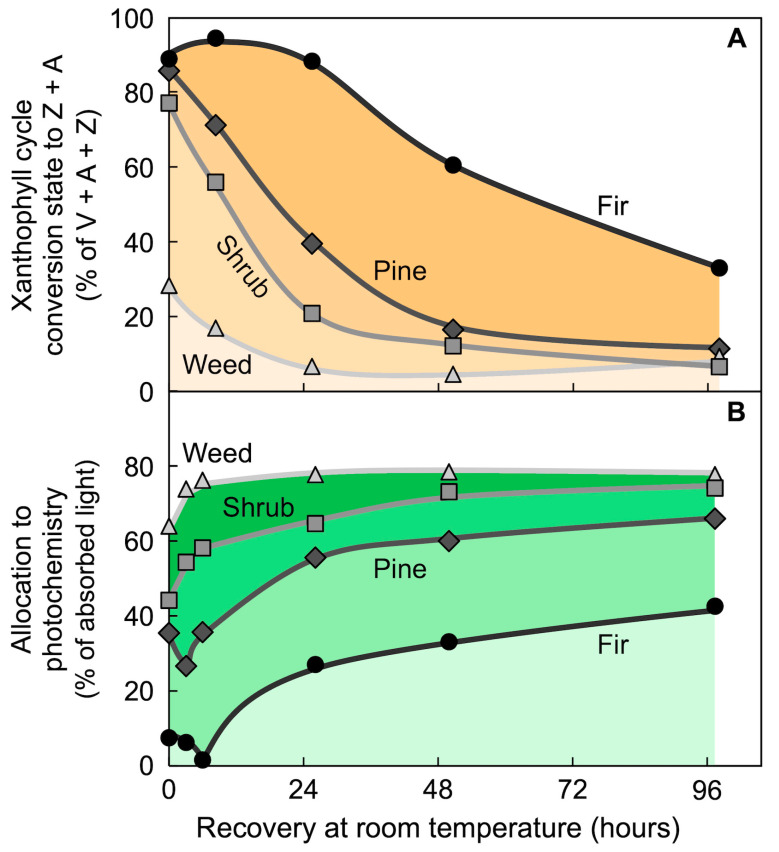
Changes in (**A**) percent of the xanthophyll cycle pool present as zeaxanthin and antheraxanthin (Z + A) and (**B**) percentage of absorbed photons allocated to photochemistry (quantified from chlorophyll fluorescence as photosystem II efficiency) in leaves of an herbaceous weed (*Malva neglecta*, light gray triangles), a woody shrub (*Euonymus kiautschovicus*, medium gray squares), and two conifers (ponderosa pine, dark gray diamonds, and Douglas fir, black circles) upon transfer from fully sun-exposed sites in mid-winter to continuous low light (10 µmol photons m^−2^ s^−1^) at room temperature. V = violaxanthin. Data are from Verhoeven et al. [93].

Figure 16 shows the relaxation kinetics of xanthophyll cycle conversion state and the percent of absorbed light available for utilization in overwintering leaves of different species upon transfer from cold field to warm laboratory conditions. An herbaceous, biennial weed that grows only in locations where photosynthesis and some level of growth can be maintained throughout the winter quickly decreased xanthophyll conversion to Z + A and increased the percentage of absorbed light available for utilization in photosynthesis when transferred to warm laboratory conditions on a cold winter day [93]. A similar response was exhibited by an overwintering evergreen shrub (cf. Figure 15; [93]). In contrast, two overwintering conifers exhibited slower relaxation kinetics of both parameters (see also [34]). Such a response may be associated with a growth arrest pattern in some species, which can be considered an adaptation to extreme environmental conditions. These findings show that, despite widely varying relaxation kinetics, close correlations were often seen in overwintering leaves between continuous maintenance of high levels of Z + A and a state of low conversion of absorbed light to photochemistry.

### 6.7. Environmental Factors other than Light Can Dominate the Response: Seasonal Heat/Aridity

**Figure 17:**
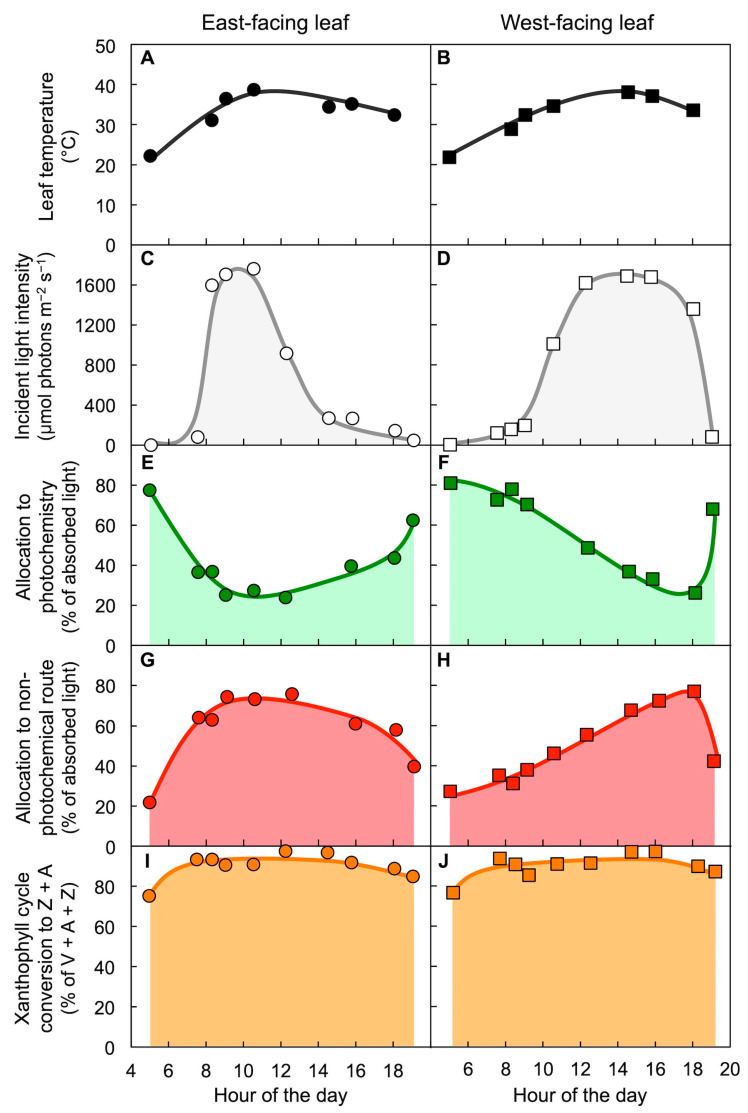
Diurnal characterization of (**A**,**B**) leaf temperature, (**C**,**D**) light incident on the upper leaf surface, (**E**,**F**) percentage of absorbed photons allocated to photochemistry (quantified from chlorophyll fluorescence as photosystem II efficiency), (**G**,**H**) percent of absorbed photons allocated to the regulated non-photochemical route (derived from maximal photosystem II efficiency as quantified via chlorophyll fluorescence), and (**I**,**J**) percent of the xanthophyll cycle present as zeaxanthin and antheraxanthin (Z + A) in east-facing (**A**,**C**,**E**,**G**,**I**) or west-facing (**B**,**D**,**F**,**H**,**J**) leaves of *Yucca brevifolia* growing in the Mojave desert on a hot day during summer. V = violaxanthin. Data are from Barker et al. [45].

Figure 17 shows the response for another evergreen that can persist through extremely hot, dry summers [45]. Despite these stressful conditions, neither east-facing leaves exposed to strong early-morning sun nor west-facing leaves exposed to strong late-afternoon sun (Figure 17A–D) maintained the photochemical system in a state with low availability of absorbed light for use in photochemistry (Figure 17E,F). At the same time, xanthophyll cycle conversion to Z + A was continuously maintained at near maximal levels day and night (Figure 17G,H). This response is different from that seen under cold-temperature stress (see Figure 15 and Figure 16), but is reminiscent of the uncoupling of xanthophyll cycle conversion state and photochemical efficiency in understory leaves acclimated to fluctuating light environments (see Figure 10 and Figure 11). It is possible that the role of zeaxanthin in membrane stabilization—relative to its role in the dissipation of excess light through the regulated non-photochemical route—is particularly critical at hot temperatures where membrane fluidity can rise to dangerous levels [65,94,95,96]. The existence of two separate control mechanisms (biochemical zeaxanthin formation and biophysical engagement of dissipation of excitation as thermal energy; see Figure 12) may thus allow maintenance of continuously high levels of zeaxanthin as a membrane stabilizer without concomitant lasting depressions in the fraction of absorbed light that can be utilized for photochemistry under conditions where plants experience heat and drought (see also [38,97]).

## 7. Conclusions

Plants’ ability to convert sunlight into photochemistry, and to handle sunlight safely over an extreme range of environmental conditions, is key to the function of the biosphere and human society. Our summary aims to illustrate the ubiquity of xanthophyll cycle-based photoprotection and the striking variety found in nature with respect to (i) xanthophyll cycle pool size, (ii) degree and speed of interconversion of its components, (iii) degree as well as onset and relaxation kinetics of excitation energy dissipation as thermal energy, and (iv) flexibility in the association among xanthophyll cycle conversion state, thermal dissipation of excitation energy, and the fraction of absorbed light available for utilization in photochemistry. It appears that the different components of this system can be independently tuned with a high degree of flexibility to produce a fit for different environments with various combinations of light, temperature, and other factors. In addition, the role of genetic variation is apparent from the variation in the response of different species growing side-by-side in the same environment. The results summarized here illustrate how field studies can generate insight into the adjustable levers that allow the xanthophyll cycle-based photoprotective system to support plant photosynthetic productivity and survival in environments with unique combinations of different factors. The advantage Yamamoto [17] contemplated of a system with multiple, independently adjustable components would thus appear to lie in its ability to offer photoprotection customized for just about any environment. Future research is needed to further elucidate the mechanisms (see, e.g., [37,80,81,83,85,88,90,98,99,100]) that provide these additional layers of control for the operation of the xanthophyll cycle and regulated non-photochemical dissipation of excitation energy. Of particular interest are mechanisms underlying the wide range of variation in the kinetics of zeaxanthin reconversion to violaxanthin and disengagement of regulated non-photochemical dissipation. Such studies should be undertaken in whole plants fully acclimated to the study conditions rather than in excess light-naïve plants. Finally, a better understanding of the levers that allow uncoupling of zeaxanthin accumulation and retention from the dissipation of excitation energy through the regulated non-photochemical route may serve in the co-optimization of crop yield and zeaxanthin content as an essential human micronutrient (see [31]).
